# The Applications of Solid-State NMR to Conducting Polymers. The Special Case on Polyaniline

**DOI:** 10.3390/molecules25030444

**Published:** 2020-01-21

**Authors:** Zoran Zujovic, Paul A. Kilmartin, Jadranka Travas-Sejdic

**Affiliations:** 1 Polymer Electronics Research Centre and the NMR Centre, School of Chemical Sciences, the University of Auckland, Private Bag 92019, Auckland 1010, New Zealand; 2 Polymer Electronics Research Centre, School of Chemical Sciences the University of Auckland, Private Bag 92019, Auckland 1010, New Zealand; p.kilmartin@auckland.ac.nz (P.A.K.); j.travas-sejdic@auckland.ac.nz (J.T.-S.); 3 MacDiarmid Institute for Advanced Materials and Nanotechnology, Victoria University of Wellington, P.O. Box 600, Wellington 6140, New Zealand

**Keywords:** solid-state NMR, polyaniline, conducting polymers, polarons, bipolarons, mechanism, emeraldine salt, conductivity

## Abstract

Polyaniline is one of the most well studied conducting polymers due to its advanced electrical, chemical, redox and morphological properties. The high conductivity of regular polyaniline, when partially oxidized and doped under acidic conditions, has been associated with the formation of unique electronic states known as polarons and bipolarons. Alternative aniline oxidation products and interesting nanotube and nanorod forms have been observed as the synthesis conditions are varied. Solid-state NMR has offered great opportunities for structural investigations and the determination of molecular dynamics in such a complex and diverse material. This review summarizes various applications of solid-state NMR techniques to polyaniline and its derivatives and the information that can be obtained by solid-state NMR.

## 1. Introduction

Although known to the scientific community for more than 150 years, polyaniline (PANI) only attracted considerable attention over the past four decades due to its electro-conductive properties and diverse and interesting chemistry. PANI is insoluble in common solvents and is relatively difficult to process, limiting the application of solution NMR for structural studies [1,2,3,4]. On the other hand, solid-state NMR (SSNMR) has been established as a very powerful analytical technique that can provide invaluable information about solid organic polymers. These include molecular dynamics, structural and conformational properties using various isotopes such as ^13^C, ^15^N and ^1^H, irrespective of the level of crystallinity and the presence of long-range order in the solid material [5,6]. Due to these characteristics, SSNMR has proven to be an invaluable technique for the analysis of PANI and various physicochemical processes the polymer can undergo.

Therefore, the applications of solid-state NMR to PANI and its derivatives will be summarized in this review. The paper consists of two main parts. In the first part, a short background to SSNMR and PANI will be presented. The second part is based on a review of SSNMR applications to PANI reported elsewhere including the detailed SSNMR analysis of three specific topics related to PANI: the application of PANI as an antioxidant, a structure of PANI nanosheets and charge carriers in nanostructured PANI.

## 2. PANI Background

Conducting polymers represent a distinctive family of organic-based polymeric materials that exhibit the electrical and optical properties of metals or semiconductors while retaining the mechanical properties and relative ease of preparation of organic polymers [1,2]. To become conductive, a polymer must possess overlapping π-molecular orbitals and a high degree of π-bond conjugation [1,2]. The π-conjugated system has alternating single and double bonds of carbon atoms along the polymer backbone. If it is in a neutral state, the conjugated polymer will act as an insulating material. However, the removal of a π-bond electron density from the conjugated polymer chain forms a radical cation defect, which is called a polaron. In this way, the electrons in these delocalized orbitals become mobile and consequently move along the polymer backbone to conduct the electrical current. The material can be “doped” by oxidation (which removes some of the delocalized electrons) or, “doped” by reduction (which adds electrons to the delocalized system). In this process, the chemicals responsible for the removal or addition of electrons to the polymeric chain are called dopants. In short, doping enables the free movement of electrons along the conjugated polymer backbone, making the polymer highly conductive [1,2]. 

One of the most researched and reported conducting polymers belongs to the century-old aniline family of polymers, namely polyaniline (PANI) [4,7,8]. There have been numerous reports on the chemical and electrochemical synthesis and applications of different forms of PANIs and their characterization in terms of electronic, magnetic, and optical properties [1,2,3,4,7,8]. Interest in PANI has rapidly increased in recent years, not only because of its electrical and optical properties but also because of its ability to form very well organized structures at the nano- and microlevels (spheres, tubes, wires, fibers, etc.) [9,10,11,12,13,14,15,16,17,18,19,20,21,22,23]. Considering the ever-increasing interest for miniaturization of electronic devices within nano- and microtechnology, it is not surprising that nanostructured conducting polymers have attracted so much attention [2,3,4,7,8]. PANI has become one of the most valuable and extensively applied conducting polymers, with applications ranging over polymer electronics, chemical sensors and biosensors, supercapacitors, antimicrobial agents, photoelectrochemical solar cells, nerve tissue engineering, anticorrosive materials, and electromagnetic interference shielding [3,4,7,8]. PANI is unique among the inherently conducting polymers as its conductivity can be reversibly controlled either electrochemically (by oxidation/reduction) or chemically (by protonation/deprotonation) [24,25]. 

**Scheme 1:**
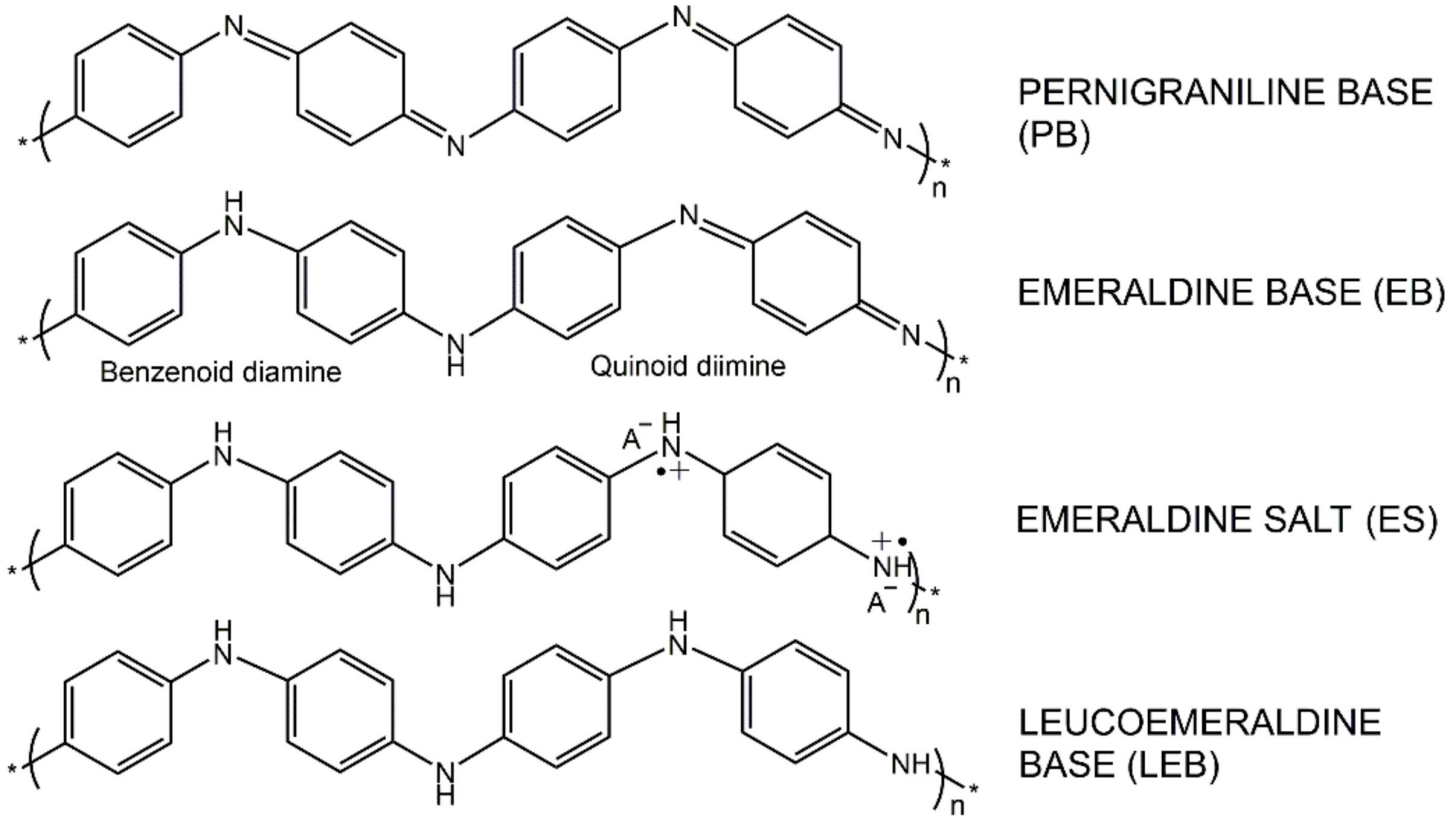
Oxidation states of polyaniline (PANI). Adapted with permission from reference [26]. Copyright (2018) John Wiley and Sons.

**Scheme 2:**
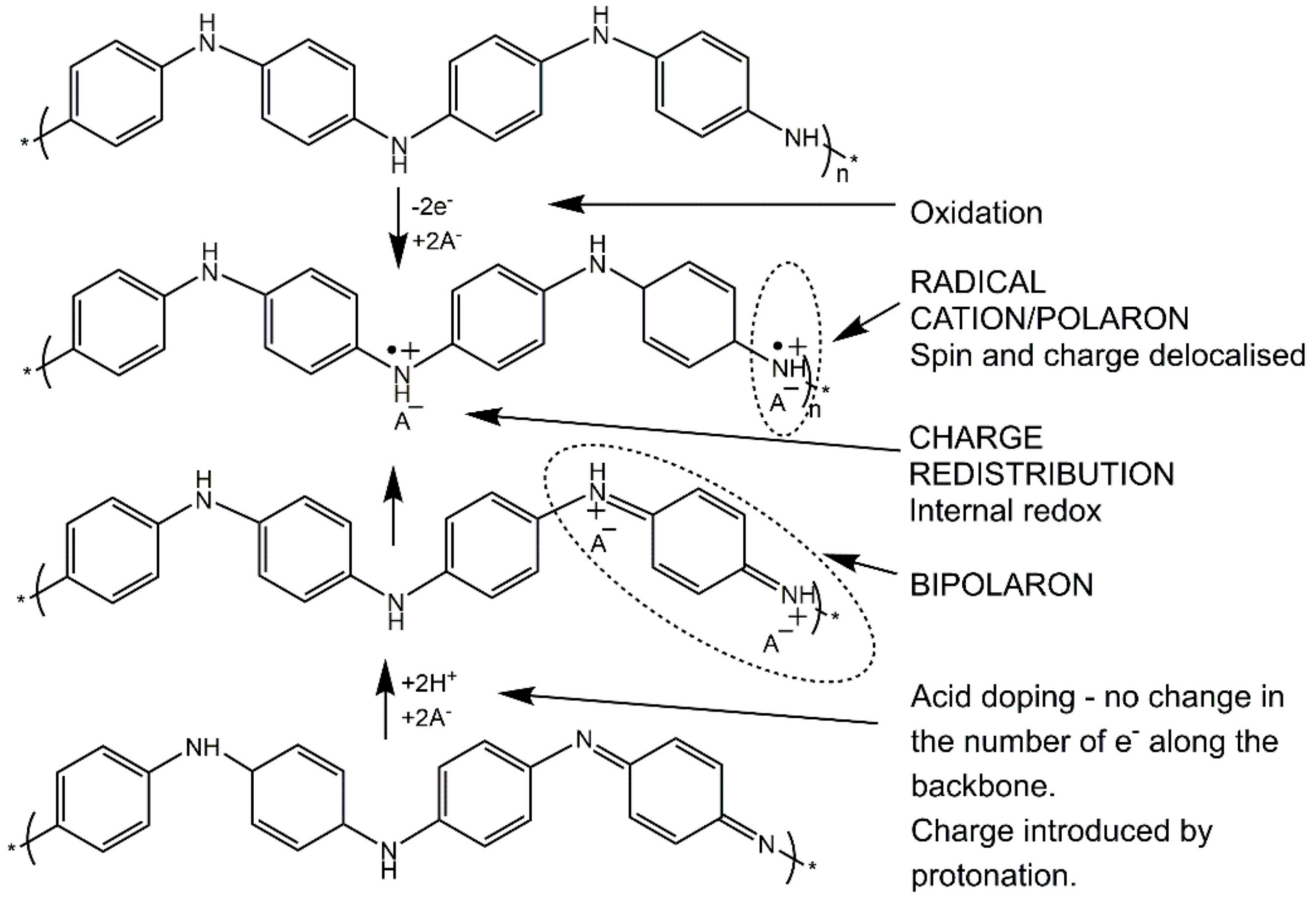
The oxidative and chemical doping routes resulting in the doped emeraldine salt (ES) state. Adapted with permission from reference [26]. Copyright (2018) John Wiley and Sons.

There are three distinct oxidation states of PANI, as shown in Scheme 1: the fully oxidized pernigraniline base, PB (insulating) that contains quinoid/imine units, the partially oxidized emeraldine base, EB (insulating) that contains both quinoid/imine and benzenoid/amine units and its doped emeraldine salt, ES (conductive form), and the fully reduced leucoemeraldine base, LEB (insulating), consisting only of benzenoid/amine units. The electroactive polymer can occur in a range of different oxidation states, determined by the ratio of amine to imine nitrogen atoms, i.e., the benzenoid to quinoid alternating segments:

One of the unique characteristics of the products obtained in the oxidative polymerization of aniline is that different types of conducting states can be obtained by both oxidation as well as chemical doping. This intrinsic feature is due to the existence of the amine groups in the backbone of the polymer, as shown in Scheme 2 [26].

## 3. SSNMR Application to PANI Background

### 3.1. ^13^C Cross-Polarization Magic Angle Spinning (CP/MAS) Spectroscopy

The structural characterization of PANI is somewhat limited because of its insolubility in common organic solvents [1,2]. Bearing in mind this drawback, solid-state NMR can offer a promising way to explore complex systems such as PANI [12,13,14,15,16,17,18,19,27,28]. Note that throughout the paper we will use a term PANI for the dedoped, non-conductive polymer (emeraldine base, EB) and we will specifically emphasize cases of the doped and conductive form, i.e., emeraldine salt (ES), along with other non-conductive forms, i.e., leucoemeraldine (LEB) or pernigraniline (PB) base. 

**Scheme 3:**
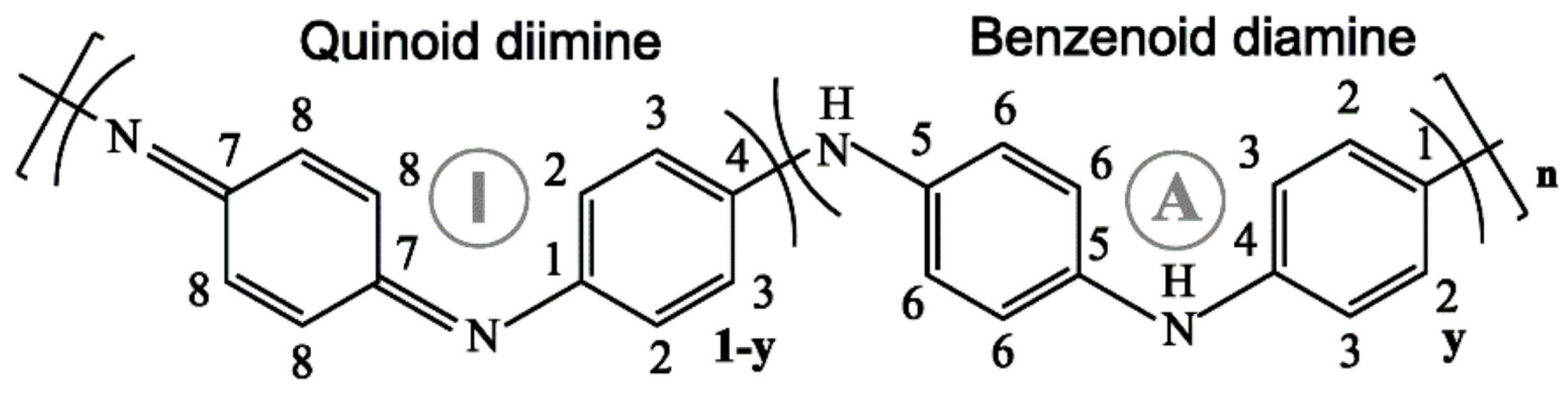
Chemical structure of PANI in the emeraldine base form.

Generally speaking, when applied to PANI, SSNMR can provide essential information regarding the structure and conformation of the polymer backbone and the nature and distribution of charge carriers [16,27]. Also, very valuable information can be obtained on the nature of dopants, structural and dynamic properties of other components added during the synthesis, and the final composition of the materials [29,30,31]. Differences in synthesis conditions lead to variations of polymer length and the presence of branched structures and non-regular aniline oxidation products. Being a multinuclear technique, SSNMR opens up possibilities for structural and dynamical investigations using various isotopes such as ^13^C, ^15^N, ^2^H, ^1^H, ^31^P, ^19^F, ^7^Li etc. [12,30,31,32,33,34]. Amongst these isotopes, ^13^C, ^1^H and ^15^N are the most valuable nuclei for the investigation of the PANI backbone structure (Scheme 3). ^13^C cross-polarization magic angle spinning (CP/MAS) spectra [6] of PANI consists of resonances which are partially overlapped, mostly due to compositional defects, differences in the sequencing of benzenoid and quinoid units, the distribution of torsion angles between adjacent rings, thermally induced molecular motions and the rotations (or flips) of benzenoid rings about their 1, 4 axes [35]. 

**Figure 1:**
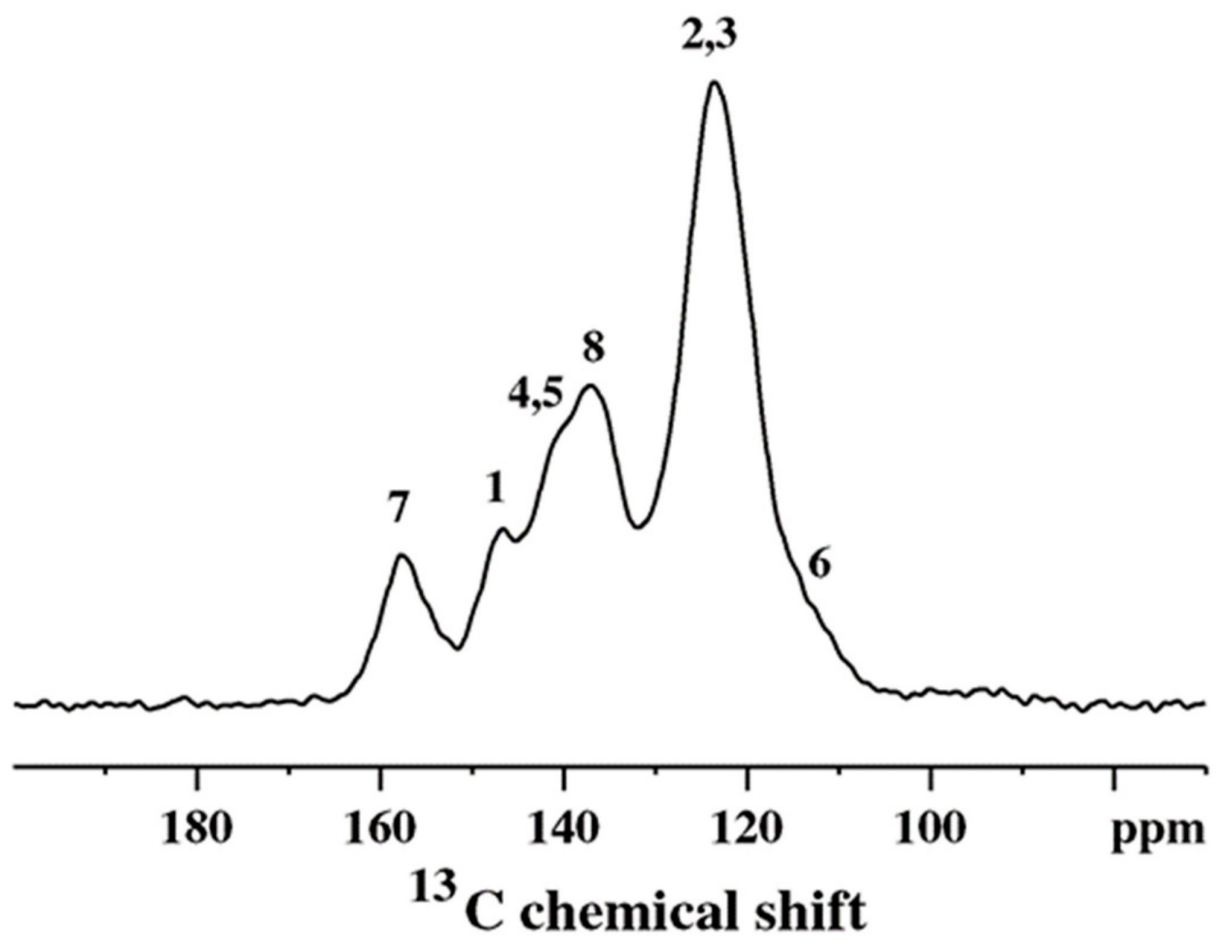
A typical ^13^C cross-polarization magic angle spinning (CP/MAS) spectrum of chemically synthesized PANI.

A typical example of a ^13^C CP/MAS spectrum of standard, chemically synthesized PANI is shown in Figure 1. The PANI spectrum exhibits six relatively distinct spectral features: at 114 (shoulder), 123, 137, 141 (shoulder), 147 and 158 ppm [28]. The peak at 123 ppm and shoulder at 114 ppm are attributed to carbons C-2,3 and C-6, respectively (Scheme 3) [28]. The peak at 137 ppm originates from the protonated C-8 carbon that belongs to the quinoid part of the PANI structure as does the non-protonated C-7 peak at 158 ppm [28]. The composite peak at 141 ppm is assigned to C-4 and C-5 carbons, while the peak at 147 ppm is assigned to the C-1 nonprotonated carbons, respectively [28]. This assignment completely describes the polymer in terms of the position of various carbons along the backbone and the relative ratio of the benzenoid to quinoid segments (Scheme 3).

On the other hand, the spectrum of a conductive emeraldine salt (ES) form consists of a single, very broad and unresolved resonance. The excessive inhomogeneous broadening (~60 ppm) [35] comes from the heterogeneous nature of the ES material, due to differences in packing, conformations, etc. These cause local variations in charge density along the polymer chain, resulting in the superposition of many, overlapping narrow resonances. The ES can be converted to the base form (EB) by using a base such as NH_4_OH, which removes charge carriers, and as a result, the ^13^C spectrum of the dedoped sample will eventually resemble the spectrum shown in Figure 1.

### 3.2. ^15^N CP/MAS Spectroscopy

**Figure 2:**
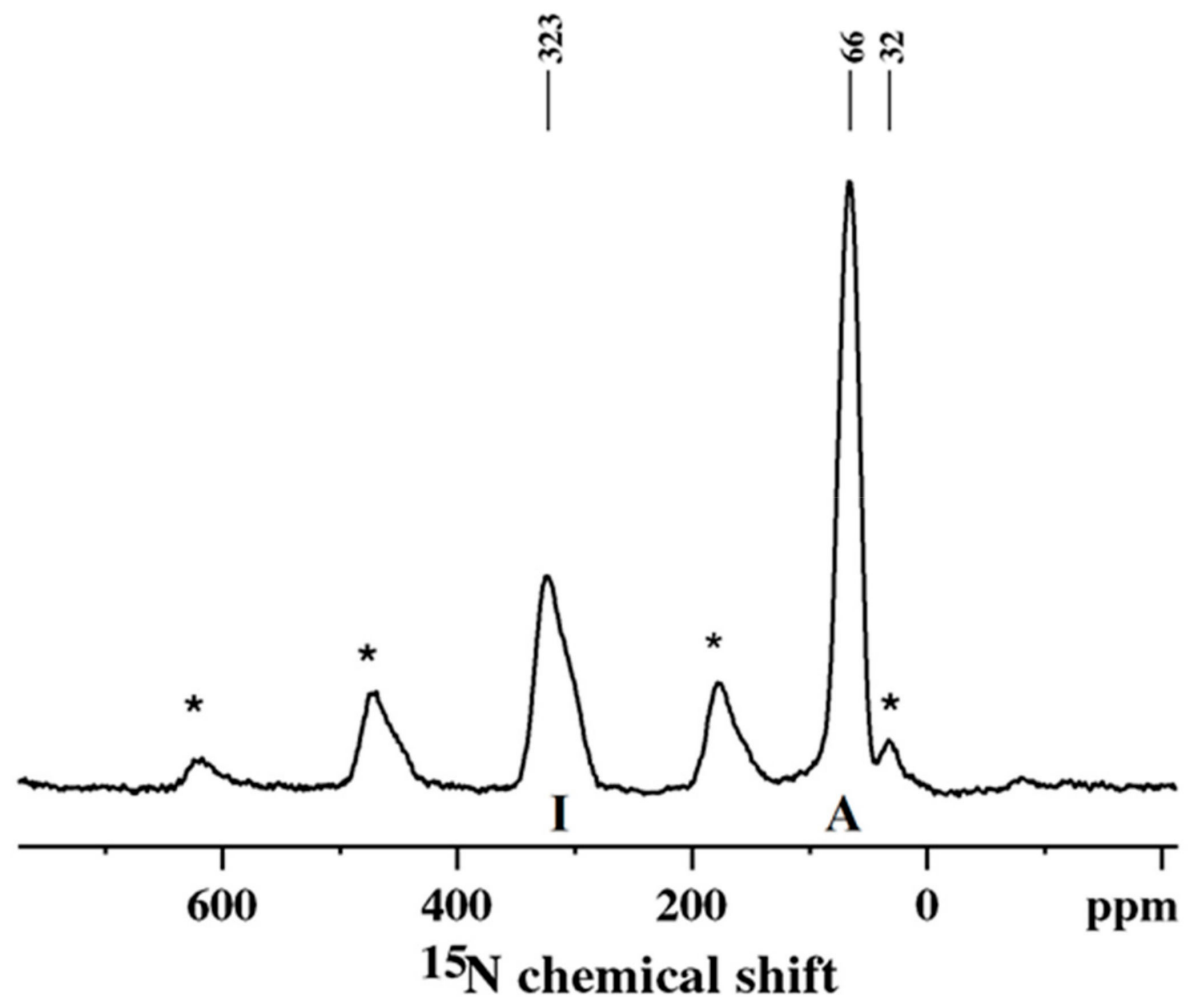
A standard ^15^N CP/MAS spectrum of PANI obtained using ^15^N-labelled aniline.

^15^N CP/MAS NMR spectroscopy of PANI obtained by the oxidative polymerization of the ^15^N-labelled monomer of aniline offers a much better insight into the sequencing of the oxidized (quinoid) and reduced (benzenoid) units along the polymer backbone [36,37]. The ^15^N spectrum consists of two well-separated peaks (Figure 2). 

These are assigned to imine (323 ppm, I) and amine (66 ppm, A) nitrogens (Scheme 3) [36,37]. The sidebands denoted by asterisks originate from the imine peak at 323 ppm. Thus, the ratio of benzenoid to quinoid units can be determined directly from the relative areas (corrected for the *T*_1ρ_^H^ and T_CH_ effects) of these peaks and the related sidebands. The peak at ca. 32 ppm, which originates from end-groups, is partially overlapped by an imine sideband. In the case of a doped PANI (ES), the imine peak will disappear, and a broad shoulder will appear upfield, in the 80–150 ppm region [36,37]. The appearance of the shoulder is due to the presence of positively charged nitrogen atoms (see Scheme 2) and variations in the charge density along the polymer chain caused by heterogeneity in PANI packing and the existence of different conformations [36,37].

## 4. SSNMR Applications to PANIs 

### 4.1. The Structure and Molecular Dynamics of PANI 

Devreux et al. applied ^13^C SSNMR to various conducting polymers, including doped and dedoped PANIs. According to the authors, this was the first NMR experimental evidence of the presence of alternating benzenoid/reduced and quinoid/oxidized rings in PANI [38]. They used the selective experiment, i.e., the NQS (non-quaternary suppressed) pulse sequence [5,6] to differentiate protonated (proton-bonded carbons) from non-protonated carbons and perform a peak assignment in the spectrum of dedoped PANI. The NQS experiment could not resolve lines in the spectrum of doped PANI [38]. 

Hjertberg et al. [39] applied ^13^C CP/MAS to investigate the structure of PANI. Based on the spectral data of model substances and their comparison to the PANI spectra, the authors confirmed the presence of a benzenoid-quinoid alternating structure in PANI. 

Hagiwara et al. was one of the first groups to study PANI using ^13^C CP/MAS experiments [40]. They assigned peaks in solid-state NMR spectra using solution NMR data obtained on model substances with well-known structures. They reported that PANI was made up of both amine and benzenoid-quinoid alternating segments, confirming the previous findings reported by Chiang and McDiarmid [25]. 

Kaplan et al. analyzed the LEB, EB, and ES hydrochloride forms of PANI using ^13^C CP/MAS experiments [35]. They also confirmed the presence of alternating quinoid and benzenoid units in the EB form of PANI. The line broadening (a linewidth of ~10 ppm) was introduced by the structural heterogeneity caused by the changes in the local conformational and configurational geometries, variations in chain packing and compositional defects. The authors excluded motional broadening as the linewidths did not depend on temperature up to 100 °C. The conductive form of PANI ES hydrochloride resulted in the extensively broad, unresolved resonance with a linewidth of ~60 ppm. To explain the rather large linewidth and to differentiate between homogeneous and inhomogeneous broadening, the authors carried out a spin-echo experiment. They showed that the homogeneous line was only a few ppm broad. Thus, the featureless/broad line, which was inhomogeneously broadened, consisted of many homogeneously broadened overlapping narrow resonances caused by the heterogeneous nature of the material. This excluded electron spin induced relaxation as a possible cause for the line broadening, as this would result in homogeneous broadening. Rather, the origin of the ^13^C line broadening in the ES hydrochloride was due to the charge density distributions along the polymer backbone and the local variations in the polymer structure, which perturbed the charge distribution and created the spectral dispersion. 

Kaplan et al. performed carbon ^13^C CP/MAS and ^2^H deuterium solid-echo experiments to probe the structure and dynamics of the ES (conductive), LEB and EB (insulating) forms of PANI [41]. The main type of motion found in these polymers was the phenyl 180° flipping. This type of motion was characteristic of amorphous regions of the polymers. Using both the deuterium NMR and carbon *T*_1_ relaxation, the authors revealed a broad distribution of correlation times for ring flips (ca. 10^−7^ to 1 s). The deuterium resonances could be deconvoluted into two components with different line shapes caused by the fast and slow ring flipping constituents. The ratio of these was used to determine a free volume available to the polymer chains. The conductive ES hydrochloride contained approximately a third as many fast flippers as compared to the EB form. This was mainly because of the steric interactions with the chloride counterions and the π character of the ring-nitrogen bonds in the ES.

Kaplan et al. employed ^13^C and ^2^H solid-state NMR spectroscopy to investigate PANI in emeraldine base (EB), leucoemeraldine (LEB), and emeraldine hydrochloride (ES) forms [42]. They confirmed the presence of alternating reduced and oxidized units in the EB structure by comparing the ^13^C CP/MAS spectrum of EB to the spectra of the pristine and partly air-oxidized LEBs. The deuteron spectra obtained by the two pulse quadrupole echo experiments were used to get more information about the nature and dynamics of the major molecular (chain) motion in the temperature range from 300 to 400 K. It was found that this motion consisted of 180° ring flips about the 1, 4 axes. This motion became partly inhibited in the ES form due to the π-character of the ring-nitrogen bonds and steric interactions with the neighboring chloride counterions. At higher temperatures free volume in polymer increased, making rings sterically less constrained by neighboring chains so that the number of fast flipping rings increased [42]. 

Richter et al. used ^15^N CP/MAS to probe the LEB and EB forms of PANI [43]. They confirmed that the EB polymer consisted of alternating oxidized and reduced units, but could not detect the end-group -NH_2_ units. The authors attributed this to the low number of short-chain oligomers present in the investigated materials. 

Stein et al. analyzed data obtained by relaxation measurements (*T*_1_^C^, *T*_1ρ_^H^ and *T*_CH_) [5,6] at different temperatures (225–335 K) and pH values to show that conductive PANI was less mobile compared to its non-conductive counterpart [44]. The proton *T*_1ρ_^H^ obtained from the variable contact time experiments [5,6] was ~6 ms for the metallic/conductive PANI and ~0.8 ms for the insulating material. The authors explained this by the rapid bipolaron motion, which caused moving of relatively rigid imine groups up and down along the polymer backbone. The authors could not detect the Knight shift or, according to them, there was no electron spin density on carbons implying the bipolaron model for conduction in PANI ES [44].

Stein et al. applied a two-dimensional chemical exchange ^13^C NMR experiment on PANI [45]. This experiment offered the possibility for calculating the ratio of freely moving rings (fast flippers) to the rings that are “fixed” in one place. 

Adams et al. used ^15^N CP/MAS to determine the molecular weights of low- and high molecular weight PANIs [46,47]. They detected 1° (primary) amine groups in the ^15^N spectra, which were supposed to be at one end of the polymer chain. Consequently, Adams et al. used the ratio of the 2° (secondary) to the 1° (primary) amine groups to determine the molecular weight of PANI and compare results to data obtained by Gel Permeation Chromatography measurements. They calculated the ratio of 2° to 1° (end group) amine structural units using ^15^N peak areas and reported that that ratio for low molecular weight PANI was 14:1, and ca. 30:1 for high molecular weight PANI. The authors presented the ^15^N CP/MAS spectrum of the ES, suggesting that the protonation of the EB occurred on the imine nitrogen, which caused the imine resonance to shift upfield to 102 ppm, and become a shoulder on the 2° amine peak [46]. 

Sahoo et al. used ^13^C and ^15^N NMR to analyze PANIs obtained from peroxidase-catalyzed polymerization of aniline with and without template poly(vinylphosphonic acid) (PVP) [48]. The spectra showed that PANI obtained without a template during enzymatic polymerization resulted in the presence of branched structures with the C–C or C–N–C couplings at the 1, 2- and 1, 4- positions. This was confirmed by shifting the imine peak from 330 ppm (seen in the spectrum of a template-assisted PANI) to 275 ppm due to hydrogen bonding between imine and amine nitrogens in the branched segments. On the other hand, enzymatically synthesized PANI in the presence of a template showed predominantly the 1, 4-coupling of aniline structural units. This was confirmed in the ^15^N spectrum as the position of the imine peak was at 330.6 ppm, which was characteristic of the imine resonances detected in the spectra of standard PANIs. 

Namgoong et al. applied ^13^C CP/MAS to analyze the structure of PANI synthesized by self-stabilized dispersion polymerization [49]. They suggested that PANI obtained in this way consisted mainly of phenylene rings coupled by para-linked nitrogens with a lower density of defects compared to the conventionally synthesized PANI, which was evidenced by the ^13^C CP/MAS spectrum. Ramamurthy et al. used ^13^C CP/MAS to analyze high molecular weight PANI. NMR data supported the predominance of 1,4- coupling i.e., the prevalent presence of the linear chains [50]. 

Bláha et al. used ^13^C CP/MAS experiments to understand the effect of the p-benzoquinone on the structural characteristics of PANI obtained by the oxidative polymerization of aniline hydrochloride in the presence of ammonium peroxydisulfate as an oxidant [51]. By comparing the ^13^C CP/MAS spectra of the PANI bases prepared with varying concentrations of p-benzoquinone to the spectrum of the 2,5-dianilino-p-benzoquinone, the authors revealed the effects of p-benzoquinone on the PANI structure. 

### 4.2. The Role of Different Dopants

Raghunathan et al. used para-toluene sulfonic acid (PTSA) and sulfosalicylic acid (SSA), to dope PANIs, otherwise prepared by chemical and electrochemical methods. They investigated the structure of protonated products by using ^13^C CP/MAS [52]. The authors correlated the concentration of dopants to the crystallinity of conductive ES PANI and suggested that conduction occurs in the inhomogeneous areas consisting of “metallic” polymer particles immersed in an insulating framework.

Kawahara et al. applied ^1^H solid-state NMR measurements to explore the effects of various dopants HClO_4_, H_2_SO_4_, and H_3_PO_4_ on the electrical conductivity of PANI. The linewidths in ^1^H MAS NMR spectra were affected by the distribution of various chain domains and/or dopants in the polymer framework. Also, using the ^1^H MAS NMR spectra it was possible to show that the ratio of -NH^+^ = and -NH_2_^+^- sites was greater in the polymers doped at the higher acid concentration. The presence of narrow ^1^H NMR lines suggested the effects of the fast benzene rings motion in the polymer. It was proposed that multivalent dopants, i.e., H_2_SO_4_ and H_3_PO_4_ were forming polymer blocks which hindered electrical conductivity in the PANIs [53]. 

Yoo et al. synthesized water dispersable PANIs which were templated with a polymer poly(2-acrylamido-2-methyl-1-propanesulfonic acid, or PAAMPSA, at different molecular weights [54]. They found that the conductivity increased with a decrease in the template molecule weight, meaning that the conductivity could be controlled by the molecular weight of PAAMPSA. The authors used ^13^C CP/MAS and ^15^N CP/MAS to get more information about the structural changes of PANI due to the presence of a template. The data from ^13^C spectra implied that in the presence of a PAAMPSA template, para-coupling was dominated during the polymerization creating the linear PANI chains with the low concentration of defects. Using ^15^N CP/MAS authors were able to link the backbone structure around the amine and imine nitrogens to the conductivity. They suggested that the higher conductivity of PANI–PAAMPSA was due to the greater charge delocalization [54].

Young et al. investigated the acid distribution in the *tert*-butylphosphonic acid (TBPA) doped PANI by ^13^C and ^31^P solid-state NMR [31]. The acid distribution was revealed by analyzing ^31^P-^31^P dipolar coupling and the ^31^P-^31^P internuclear distances determined by the dipolar recoupling technique DRAMA (Dipolar Recovery at the Magic Angle). This provided information on the heterogeneous distribution of the acid molecules in the TBPA doped PANI. The DRAMA experiment suggested that at low doping levels the TBPA molecules were not randomly distributed, but rather they were placed in certain domains that were fully doped/conducting. At the same time, there were other completely undoped/insulating regions. The ^13^C CP/MAS data showed that PANI was completely doped at the TBPA:PANI ratio of 2.

Espe et al. used ^1^H-^13^C, ^1^H-^19^F and ^1^H-^15^N CP/MAS, REDOR (Rotational Echo DOuble Resonance), DRAMA and SEDRA (Simple Excitation for the Dephasing of Rotational Amplitudes) NMR experiments to explore packing in amorphous parts of HF doped PANI [30]. The authors suggested that only one-third of the signal was detected in the fully HF doped PANI. They explained this by assuming that the whole crystalline domain and a part of the amorphous domain within 50 Å of the crystalline boundary were practically undetectable as a consequence of the presence of paramagnetic delocalized electrons in the crystalline parts. The ^19^F NMR spectrum of doped PANI exhibited three chemical-shift environments which were assigned to the three possible arrangements of F^-^ counter anions in the vicinity of charged nitrogens. 

Kolbert et al. analyzed uniformly ^13^C enriched PANI doped with camphor sulphonic acid. The authors showed that the ^13^C spin-lattice relaxation rates followed a modified Korringa relation for relaxation due to the hyperfine coupling to conduction electrons [55]. This was convincing confirmation for a metallic state in doped PANI.

### 4.3. The Effects of Temperature

Kuroki et al. used ^13^C and ^15^N NMR to analyze a process of PANI carbonization [56], whereby very interesting and potentially useful material was made that could be used for catalyst supports, electrochemical catalysts, hydrogen storage etc. ^13^C and ^15^N CP/MAS spectra of the heat-treated PANIs at various elevated temperatures (200–1000 °C) in nitrogen atmosphere were presented. The authors reported a detailed scheme of the structural changes in PANI during the heating. Based on the NMR spectra, it was found that the thermal reconstruction of the polymer backbone started between 400 and 600 °C. Specifically, the ^15^N CP/MAS spectra suggested that the amine and imine nitrogens converted into pyrrolic and pyridinic nitrogens, respectively. The authors performed chemical shielding calculations to support experimental data. 

Mathew et al. used ^13^C and ^15^N CP/MAS to analyze the reactivity, structure and oxidation state of PANI in *N*-methyl-pyrrolidinone solutions heated over the temperature range 120−190 °C [57]. They showed that the polymer went through a combination of cross-linking and reduction (being fully reduced after heating at 190 ℃ for 2 h) and went through partial re-oxidation in those regions that were not cross-linked when oxygen gas was bubbled through the solutions after heating. The ^15^N NQS (interrupted decoupling) experiments [5,6] of the PANI films cast from heated *N*-methyl-2-pyrrolidinone (NMP) solutions revealed the presence of phenazine units formed by cross-linking. The authors compared annealed PANI with the leucoemeraldine base (LEB) form of PANI. This observation was supported by the ^13^C and ^15^N CP/MAS spectra. The ^13^C spectrum of the sample heated at 190 °C showed the absence of the imine carbon peak at 158 ppm implying the full reduction of the polymer. The broader resonances in the spectrum of the heated sample compared to pristine LEB, suggested a greater heterogeneity caused by heating. In addition, the imine peak in the ^15^N spectrum of the sample heated at 190 °C was absent, in line with the ^13^C data, suggesting a complete reduction of the polymer [57]. 

Kuroki et al. carried out ^1^H -^15^N CP/MAS, ^15^N spin-echo, as well as *T*_1ρ_^H^ measurements to characterize pyrolyzed metal PANI cathode catalysts for oxygen reduction in fuel cells. They studied oxygen reduction reaction activity of pyrolyzed metal-free and metal (Mn, Fe, Co, Ni and Cu)-containing PANI in a polymer electrolyte fuel cell [58].

### 4.4. PANI Based Materials and Their Applications

Hasegawa et al. used solid-state NMR to explore the molecular dynamics of a PANI/β Cyclodextrin inclusion complex (PANI/β-CD IC) [59]. They synthesized a complex in which single polymer chain (PANI) entered into certain cyclic molecules β Cyclodextrin, (β-CD) and formed a necklace-like structure. The UV spectrum of PANI in the complex showed the blue-shift of 10 nm of the π-π* peak. To explore this effect, the authors carried out variable temperature ^13^C CP/MAS experiments, *T*_1C_ spin-lattice relaxation time measurements (Torchia method) and the *T*_1*ρ*_^C^ rotating-frame relaxation experiments. *T*_1C_ values would mostly reveal the twisting motion around the molecular axis, while *T*_1*ρ*_^C^ value was affected by molecular dynamics around the spin-lock frequency (in this case, 80 kHz) and would correspond to the molecular dynamics all over the chain. Due to the formation of the complex, the local rapid twisting motion around the PANI axis was facilitated, which was revealed by the *T*_1C_ measurements. At the same time, the *T*_1*ρ*_^C^ measurements showed that the inclusion by β-CD did not affect the slow-motion over the polymer chain. Thus, the inclusion resulted in the blue shift of the *π*-π* transition peak of PANI probably due to the removal of the intermolecular π-π stacking interaction and the improvement of associated wide-angle twisting motion. 

Holland et al. used ^7^Li NMR under the MAS and static conditions to investigate the local lithium environment in electrochemically lithiated nanocomposite materials which consisted of V_2_O_5_ and different PANI derivatives [60]. The spectra were presented for the V_2_O_5_ xerogel, PANI/V_2_O_5_, and PSPAN/V_2_O_5_ (PANI N-propane sulfonic acid/V_2_O_5_) materials which incorporated different amounts of ion-exchanged and electrochemically intercalated Li^+^. The NMR experiments demonstrated that Li^+^ was presented in the ion-exchange sites of V_2_O_5_ xerogels and PSPAN/V_2_O_5_ nanocomposites (strong ^7^Li peak at ~0 ppm). On the other hand, for PANI/V_2_O_5_ nanocomposites the ion-exchanged Li^+^ was almost completely absent (minor ^7^Li peak at ~0 ppm). These results were explained by the charge compensation based on the structures of the PANI/V_2_O_5_ and PSPAN/V_2_O_5_. 

Goward et al. analyzed polymer/transition metal oxide nanocomposites, specifically, PANI interleaved between the layers of MoO_3_ [61]. They used the quadrupole echo ^2^H NMR experiments to investigate the conformation and molecular dynamics of perdeuterated PANI located between the layers of MoO_3_. The ^2^H NMR data provided invaluable and convincing evidence about the preferential orientation of PANI in the MoO_3_ host framework. 

Shi et al. used the in situ nano-assembly of bacterial cellulose (BC) and PANI to form an electroconductive hydrogel that encompasses the properties of hydrogels and conductive systems such as conducting polymers [62]. The structural features of BC and BC-PANI were investigated using the ^13^C CP/MAS experiments which confirmed the formation of the biocomposite. Specifically, the authors proposed that the hydroxyl (–OH) of C6 from BC primary alcohol group reacted via dehydration with PANI. This was evidenced in the ^13^C CP/MAS spectrum of BC-PANI by merging of the C6 resonance with the other peaks. 

Young et al. analyzed the interaction of gel inhibitors (GI) 2-methylaziridine (2-MA) and pyrrolidine (Py) with PANI using ^15^N and ^13^C CP/MAS [63]. The secondary amines as gel inhibitors serve for making stable highly concentrated PANI solutions in NMP. However, at the same time, the structure of the polymer could be somewhat changed. The SSNMR was used to reveal these changes. The data revealed that Py covalently attaches to the quinoid ring of ~50% of the alternating units converting the portion of the EB form to the LEB form. Although 2-MA slightly reacted with PANI, it was physically trapped in the film in the amount of 0.7 2-MA per PANI. The *T*_1ρ_^H^ measurements showed that in the case of the PANI/Py film the presence of the pendant amine group disrupted the chain packing which was reflected in the increased amount of kHz regime chain motion compared to that measured in the PANI/2-MA film. 

Goddard et al. used ^2^H quadrupole echo and ^2^H MAS NMR experiments to get information about the structure of PANI intercalated into montmorillonite clay layers [33]. Quadrupole echo spectra suggested the presence of different fractions of fast flipping phenyl rings for EB and ES hydrochloride. A deuteron MAS spectrum of ES revealed an additional set of spinning sidebands (SSBs) with different linewidths and intensities compared to the EB spectrum. This set of SSBs was shifted 6 ppm in comparison to the SSBs obtained in the EB spectrum. The presence of the additional set of SSBs was due to the presence of metallic regions and the deuteron nuclear spin interaction with delocalized electron spins which was manifested as a Knight shift. This observation suggested the involvement of polarons in charge transfer in the metallic regions [33]. 

Gizdavic et al. used a ^13^C CP/MAS technique to investigate poly(aniline-co-ethyl 3-aminobenzoate) copolymers [29]. To facilitate peak assignments, NQS experiments were carried out. The ^13^C CP/MAS results implied an integration of the aminobenzoic acid and ethyl 3-aminobenzoate units in the copolymer chains. This was based on the presence of the peaks at 165.0 ppm (C=O), 14.7 ppm (CH_3_) and 61.3 ppm (CH_2_) from the ethyl group in the spectra of copolymers. Furthermore, NMR data suggested that the copolymer structures primarily consisted of the benzenoid and quinoid alternating segments. 

**Figure 3:**
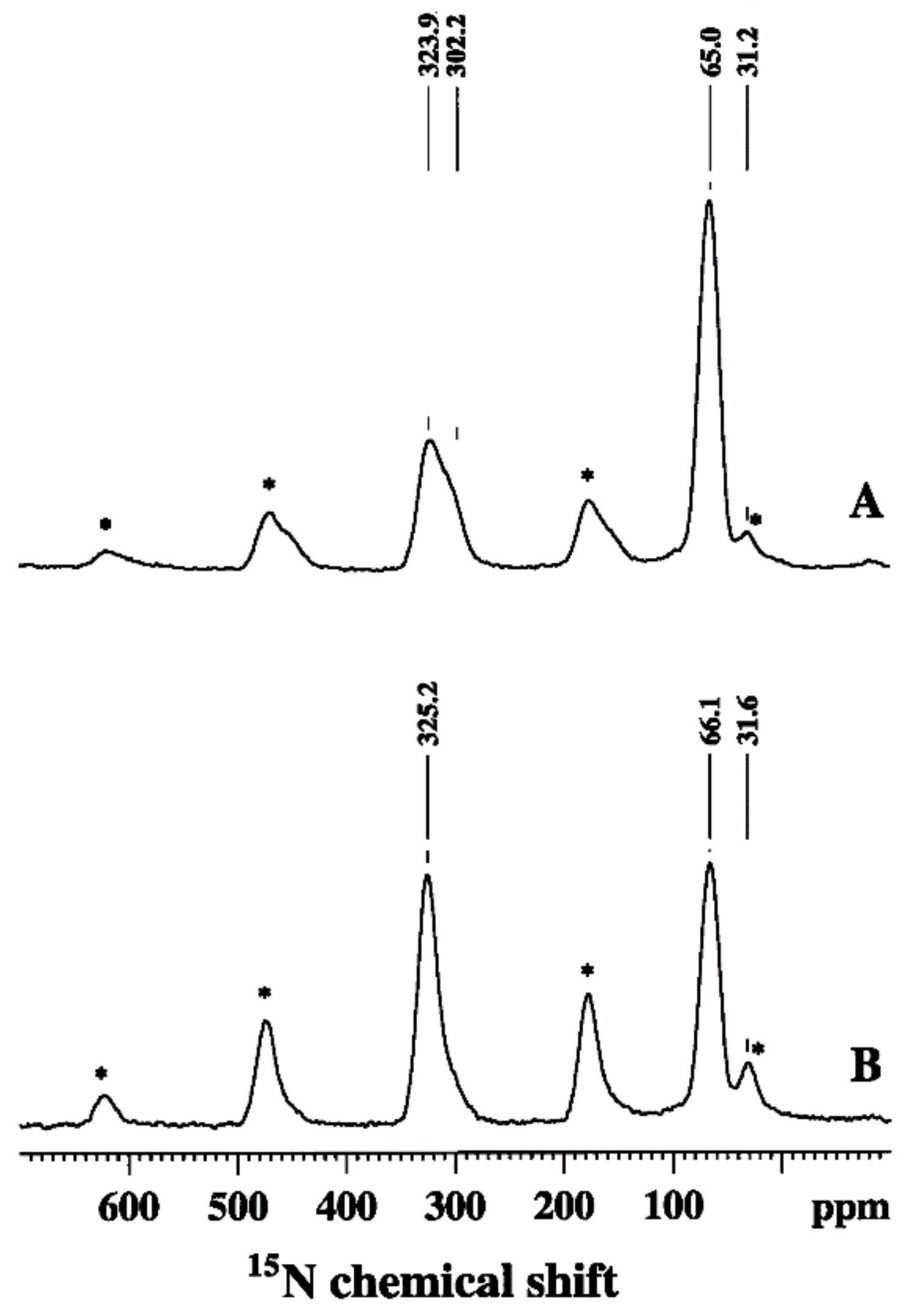
^15^N CP/MAS spectra of the 15N labeled PANI before (**A**) and after (**B**) reaction with DPPH. The asterisks denote spinning sidebands. Reprinted with permission from reference [28]. Copyright (2006) Elsevier.

**Figure 4:**
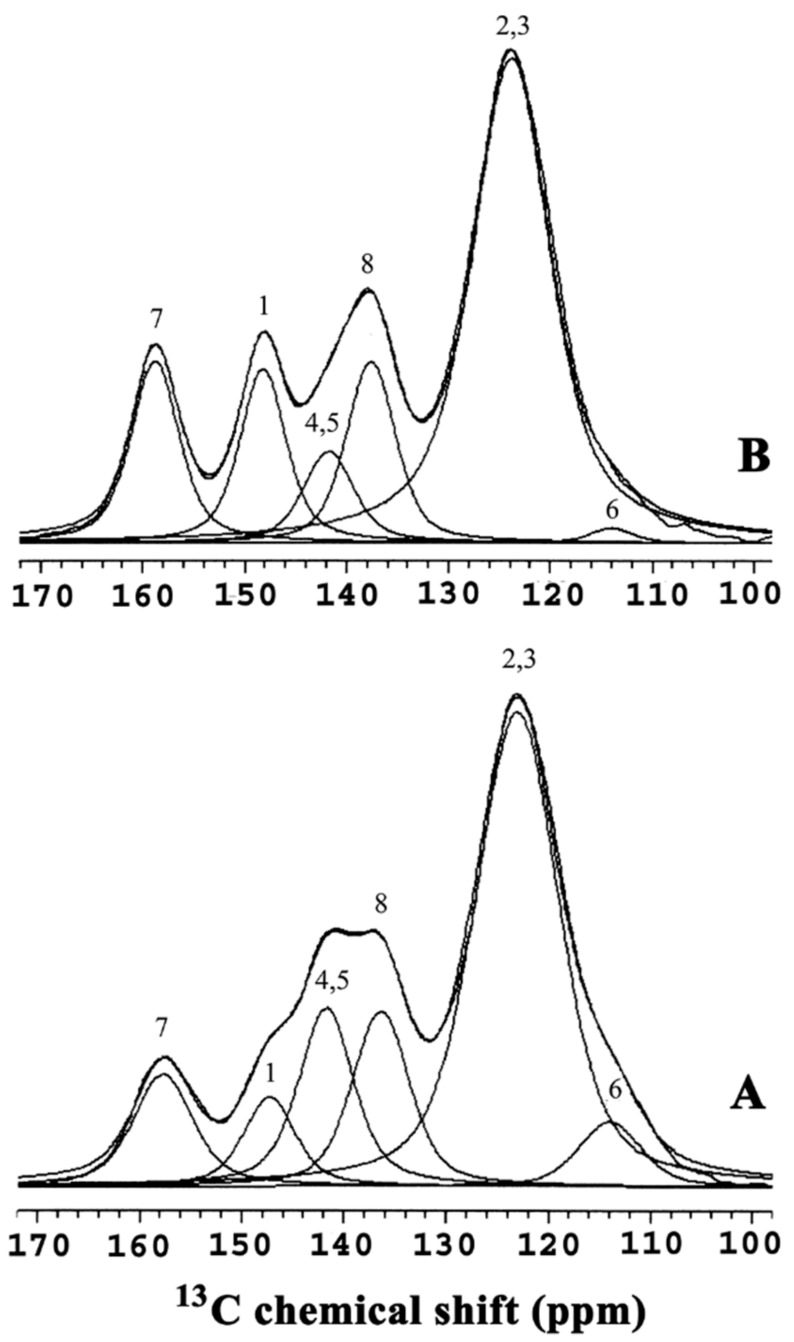
Deconvoluted ^13^C CP/MAS spectra of PANI (**A**) and PANI–DPPH (**B**). Reprinted with permission from reference [28]. Copyright (2006) Elsevier.

^13^C and ^15^N CP/MAS NMR were applied to reveal the antioxidant (radical scavenging) properties of PANI [28]. ^15^N labeled PANI was suspended in DPPH (1,1-diphenyl-2-picrylhydrazyl) radical-containing solution. Due to its antioxidant properties, PANI was oxidized in this reaction (PANI–DPPH), and this change was monitored and quantified by ^13^C and ^15^N CP/MAS and variable contact time measurements [6]. Figure 3 shows the ^15^N CP/MAS spectra obtained from the PANI before and after reaction with DPPH [28]. In the ^15^N spectrum of PANI (Figure 3A) two peaks were observed. They were assigned to imine (323.9 ppm) and amine (65.0 ppm) nitrogens [28]. The sidebands originate from the imine peak at 323.9 ppm (denoted by asterisks). The peak from the end-groups detected at 31.2 ppm was partly overlapped by an imine sideband [28]. After reaction with DPPH (Figure 3B), the imine resonance (325.2 ppm) increased in intensity relative to the amine resonance (66.1 ppm) implying oxidation of PANI [28]. To make corrections for the *T*_1ρ_^H^ and *T*_CH_ effects [6], and to obtain quantitative information regarding the state of oxidation, variable contact time experiments were carried out [6,28]. Based on the results from these experiments, Zujovic et al. found an imine to amine ratio of 1.5, compared to that of 0.8 in unreacted PANI. This suggests that around 30% of the benzenoid diamine units had been oxidized to a fully oxidized quinoid diamine [28]. This was in line with the longer *T*_1ρ_^H^ times found in the PANI–DDPH structure, implying the reduction of molecular motion, most probably because of the introduction of more rigid double bonds after oxidation [28]. The ^15^N findings were confirmed by ^13^C CP/MAS NMR results (Figure 4 and Scheme 3). The benzenoid peaks at 114 ppm (C-6) and 141 ppm (C-4,5) decreased in the PANI–DPPH spectrum [28]. Unlike the resonance at 137 ppm, which was attributed to (C-8), the resonances at 147 (C-1) and 158 (C-7) ppm which were assigned to the quinoid part, increased due to oxidation. The relative ratio for the resonances at 147 ppm (C-1) for PANI–DPPH and PANI was 1.4–1.5 (corrected for the *T*_1ρ_^H^ and T_CH_ effects) which was in line with data obtained from ^15^N experiments [28].

Kilmartin et al. used ^13^C CP/MAS to explore free radical scavenging/antioxidant properties of PANI and compare these findings to EPR data [64]. The NMR data confirmed that the PANI was oxidized upon the exposure to the DPPH (1,1-diphenyl-2-picrylhydrazyl) radical. 

### 4.5. PANI Nanostructures

The choice of an oxidant and/or acid, the concentration of the reactants, temperature and pH, will significantly determine the structural and morphological properties of the products obtained during the complex process of the oxidative polymerization of aniline [9]. Highly conductive, and structurally regular PANI polymer products were usually obtained at low pH (<2). On the other hand, a great variety of low-conductive products that exhibit very well-defined and sometimes fascinating morphologies, such as nanotubes, nanoparticles, nanoflakes, nanorods and nanospheres were obtained using higher pH synthesis conditions (>3–4) [9]. Specifically, to synthesize nanotubes, the falling-pH methodology was used [16,19]. In the falling-pH method, the aniline, the oxidant and acid were mixed in aqueous solution: the initial pH was usually 6–7 and the reaction solution was left in the fridge for 24 h for oxidation to occur. Due to the protons that were released during the oxidation of aniline, the pH of the reaction solution gradually decreased with time, during which different morphologies were formed. During the early stage of the reaction, different oligomeric products such as nanotubes, nanosheets, nanospheres, etc. were detected. Due to their precisely defined morphology self-assembled oligomeric (oligoanilinic) structures can serve either directly or indirectly as nanostructured templates for the formation of PANI nanostructures. According to one hypothesis [16], the PANI nanotubes were formed from oligoanilinic nanotubes that were initially produced in the form of thin, rolled nanosheets during the falling-pH reaction [16,65]. Later on, the reaction solution pH decreased and standard PANI started to polymerize at pH < 2 [9,65]. The final product formed at this stage represented an enclosing layer consisting of PANI polymer wrapped around smooth oligomeric nanotubes giving them a final and recognizable granular texture and tubular shape [9]. 

**Figure 5:**
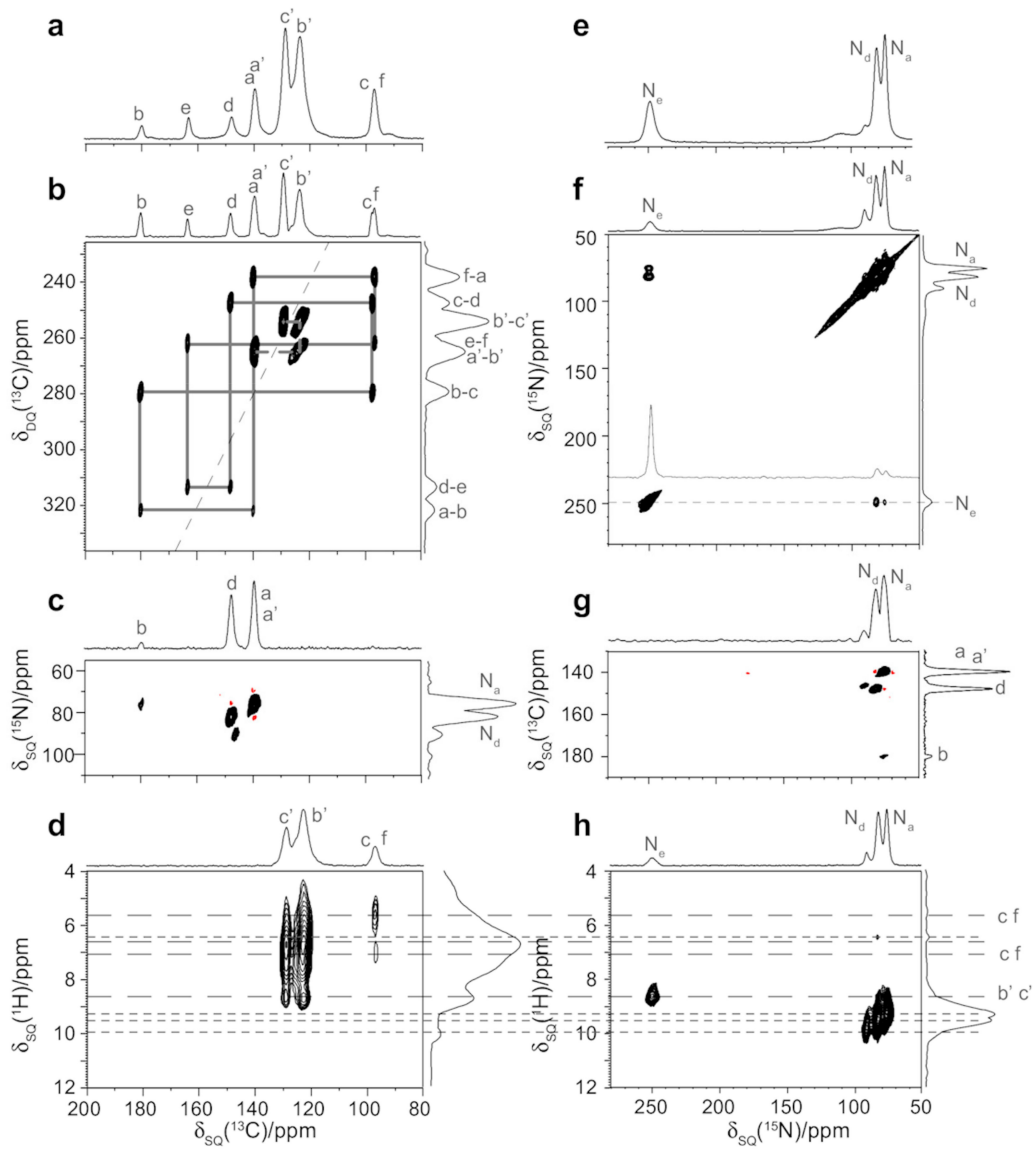
Solid-state MAS NMR (^1^H Larmor frequency of 500.1 MHz) spectra of a PANI sample synthesized using alanine and ammonium persulfate by the “falling pH” reaction: One-dimensional (**a**) ^13^C CP/MAS (20 kHz) and (**e**) ^15^N CP/MAS (12.5 kHz) spectra. (**b**) A ^13^C-^13^C refocused INADEQUATE (20 kHz MAS, 1.0 ms spin-echo duration) spectrum. (**c**,**g**) A double CP heteronuclear ^13^C-^15^N (12.5 kHz MAS) spectrum recorded with ^1^H-^15^N and ^15^N-^13^C contact times of 1.0 and 5.0 ms, respectively. In (**g**), the spectrum has been rotated through 90 degrees away from the usual convention, such that the horizontal dimension corresponds to the indirect ^15^N dimension, so as to allow comparison with the other spectra presented in the right-hand column of the Figure (**d**,**h**) Heteronuclear (**d**) ^13^C-^1^H (10 kHz MAS) and (**h**) ^15^N-^1^H (12.5 kHz) refocused INEPT spectra employing eDUMBO22 ^1^H homonuclear decoupling with spin-echo durations of (d) 0.6 and (h) 3.2 ms. (**f**) A two-dimensional ^15^N-^15^N proton-driven spin diffusion spectrum with a mixing time of 2.0 s. The spectra (**a**–**d**,**g**) were recorded on a sample synthesized starting from a 50:50 mixture of U-^13^C aniline and ^15^N-labelled aniline, while the spectra (**e**,**f**,**h**) were for a sample synthesized using ^15^N-labelled aniline. Reprinted with permission from reference [12]. Copyright (2015) American Chemical Society.

Zujovic et al. applied advanced two-dimensional solid-state NMR experiments to reveal the structure of early formed oligoanilinic morphologies obtained from a 50:50 mixture of U−^13^C aniline and ^15^N-labeled aniline [12]. The two-dimensional solid-state NMR spectra show specific carbon−carbon and carbon−nitrogen one-bond connectivities. A ^13^C−^13^C double quantum−single quantum (DQ−SQ) correlation spectrum obtained using the refocused INADEQUATE (Incredible Natural Abundance Double Quantum Transfer Experiment) [12] with a short spin−echo evolution period, 1.0 ms, ensured that correlations were only observed for directly bonded carbons (Figure 5b). Figure 5c,d shows the heteronuclear ^13^C−^15^N spectra obtained using a double CP pulse sequence [12]. An initial ^1^H−^15^N CP step creates ^15^N magnetization that evolves during *t*_1_. This was followed by a ^15^N−^13^C CP step, whereby the magnetization was transferred between dipolar-coupled pairs of ^15^N−^13^C nuclei [12]. To ensure that the correlations from both one-bond and longer range proximities were detected, a mixing time of 5 ms was used [12]. Figure 5f shows a two-dimensional ^15^N−^15^N proton-driven spin diffusion (PDSD) spectrum recorded with a mixing time of 2 s [12]. The observation of cross-peaks between ^15^N resonances at ∼80 ppm and the nonprotonated ^15^N resonance at ∼250 ppm shows that these nitrogen atoms belong to the same molecular entity [12]. The refocused INEPT24 sequence was used to reveal ^1^H−^13^C and ^1^H−^15^N one-bond connectivities (short spin−echo duration of 0.6 ms) established via through-bond J couplings (Figure 5d,h). The resolution in the ^1^H dimension was greatly improved by using eDUMBO22 ^1^H homonuclear decoupling [12]. 

**Scheme 4:**
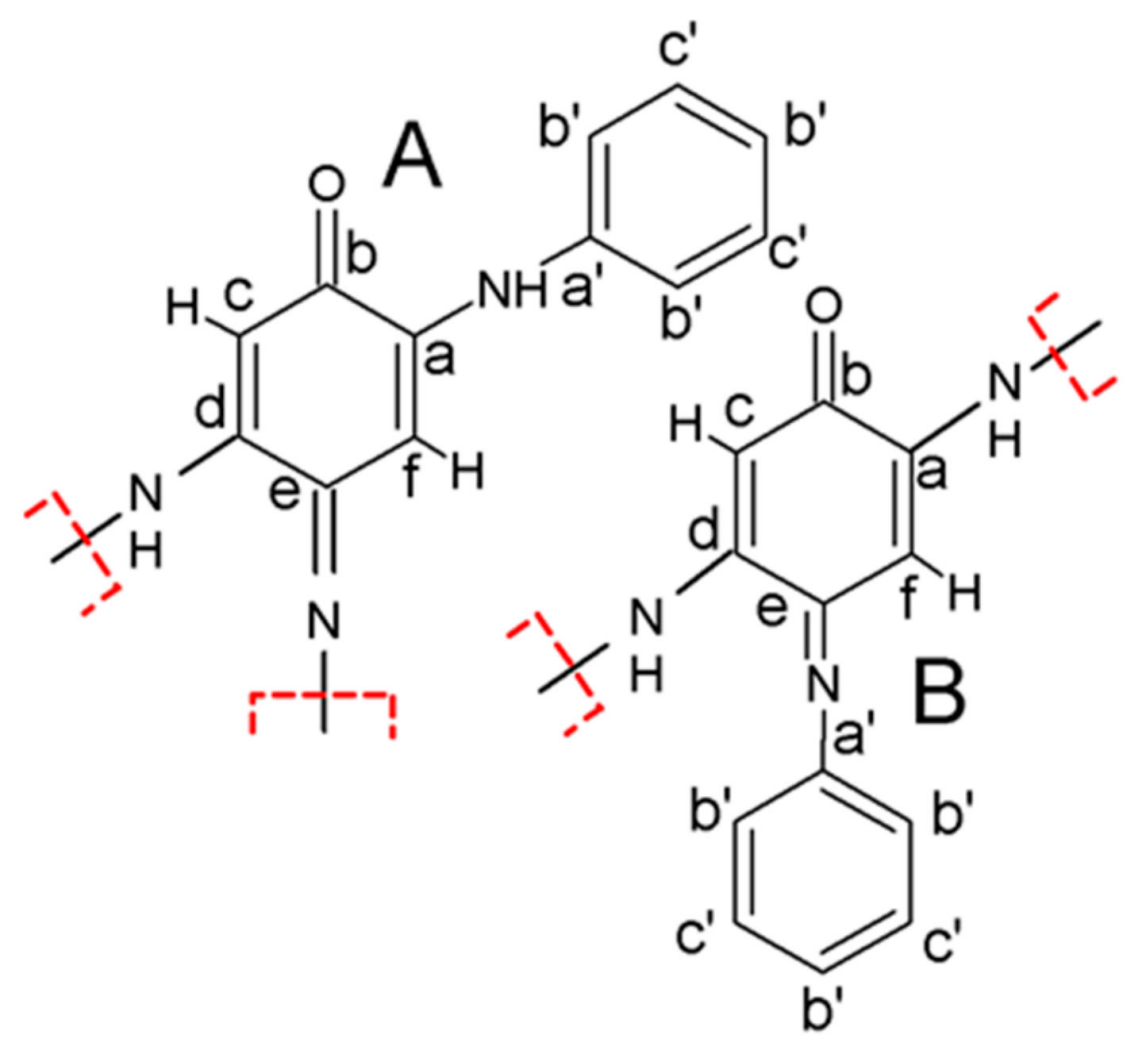
Proposed monomeric units (**A**,**B**) with red dashed lines indicating how oligomerization would proceed. Reprinted with permission from reference [12]. Copyright (2015) American Chemical Society.

Scheme 4 shows two repeating molecular units (A and B) which represent a motif for the formation of oligomeric nanosheets and nanotubes, based on the SSNMR findings. In both cases, the oligomeric backbone consists of quinoneimine units (carbons a to f), with a phenyl group (carbons a′ to c′) attached to each unit. These findings were in line with the connectivities obtained from the ^13^C−^13^C refocused INADEQUATE spectrum in Figure 5b as well as the C−N proximities detected in Figure 5c,g. The values for NMR chemical shieldings were calculated using the GIPAW approach (Gauge Including Projector Augmented Waves) [12].

Hopkins et al. compared PANI nanofibers to the standard granular form of PANI. Solid-state ^13^C and ^15^N CP/MAS NMR characterizations were able to reveal a small variation in the structural characteristics, based on the findings of two additional peaks in the ^13^C spectra (at 96.5 and 179.8 ppm) attributed solely to the nanofibers structure [66]. Also, the authors detected additional spectral features in the ^15^N CP/MAS spectrum of nanofibers (peak at 80 ppm and a sharp shoulder peak at 92 ppm) which were typically not found in the spectrum of the standard PANI. This finding, as in the case of the ^13^C spectrum, suggested to some extent different chemical environments for the nanofibers.

Zujovic et al. employed solid-state ^13^C and ^15^N NMR in the investigation of PANI nanofibers [14]. They used the CP/MAS, variable contact time and NQS techniques to reveal that the PANI nanofibers which were produced during the rapid-mixing reaction in the presence of strong acid consisted of alternating oxidized and reduced units and that they resembled the standard PANI structure. NQS experiments implied the presence of cross-linking in the nanofibers. ^15^N NMR VCT (variable contact time) measurements revealed that the ratio of the imine to amine nitrogens was 0.8, meaning that a significant portion of nanofibers remained under-oxidized [14]. 

Yang et al. studied self-doping PANI (SPANI) nanofibers and confirmed their formation by ^13^C CP/MAS experiment [67]. The SPANI nanofibers were obtained in the self-assembly process in the presence of a self-doping monomers o-aminobenzenesulfonic acid (SAN) and aniline (AN). According to the authors, the five aromatic peaks were related to the 12 aromatic carbons of the partial symmetric repeating units (AN and SAN). 

Thiyagarajan et al. used solid-state NMR to analyze enzymatically synthesized PANI nanocomposites [68] They reported that the structure of PANI nanocomposite in the as-synthesised form was similar to that of enzymatically and chemically synthesized PANI. The authors applied the ^13^C CP/MAS, variable contact time and the NQS experiment with different dipolar dephasing delays. 

Itoh et al. used ^13^C CP/MAS to study the structure and dynamic properties of layered organic-inorganic nanohybrids ((PANI)xMoO_3_ and (PoANIS)xMoO_3_) formed from MoO_3_ as a host and PANI and poly(o-anisidine) (PoANIS) as interlayer components. [69]. The incorporation and the orientation of PANI and PoANIS within the nanohybrid framework was confirmed by ^13^C CP/MAS data. 

Zha et al. used PANI for grafting onto fluorinated multi-walled carbon nanotubes (MWCNTs) and carbon nanofibers (CNFs) in toluene to get stable dispersions of functionalized nanocarbons which could be used for the fabrication of superhydrophobic films. The grafting was investigated by multinuclear ^13^C, ^1^H, ^19^F MAS and ^13^C CP/MAS solid-state NMR [70].

### 4.6. The Role of Charge Carriers in PANIs 

**Figure 6:**
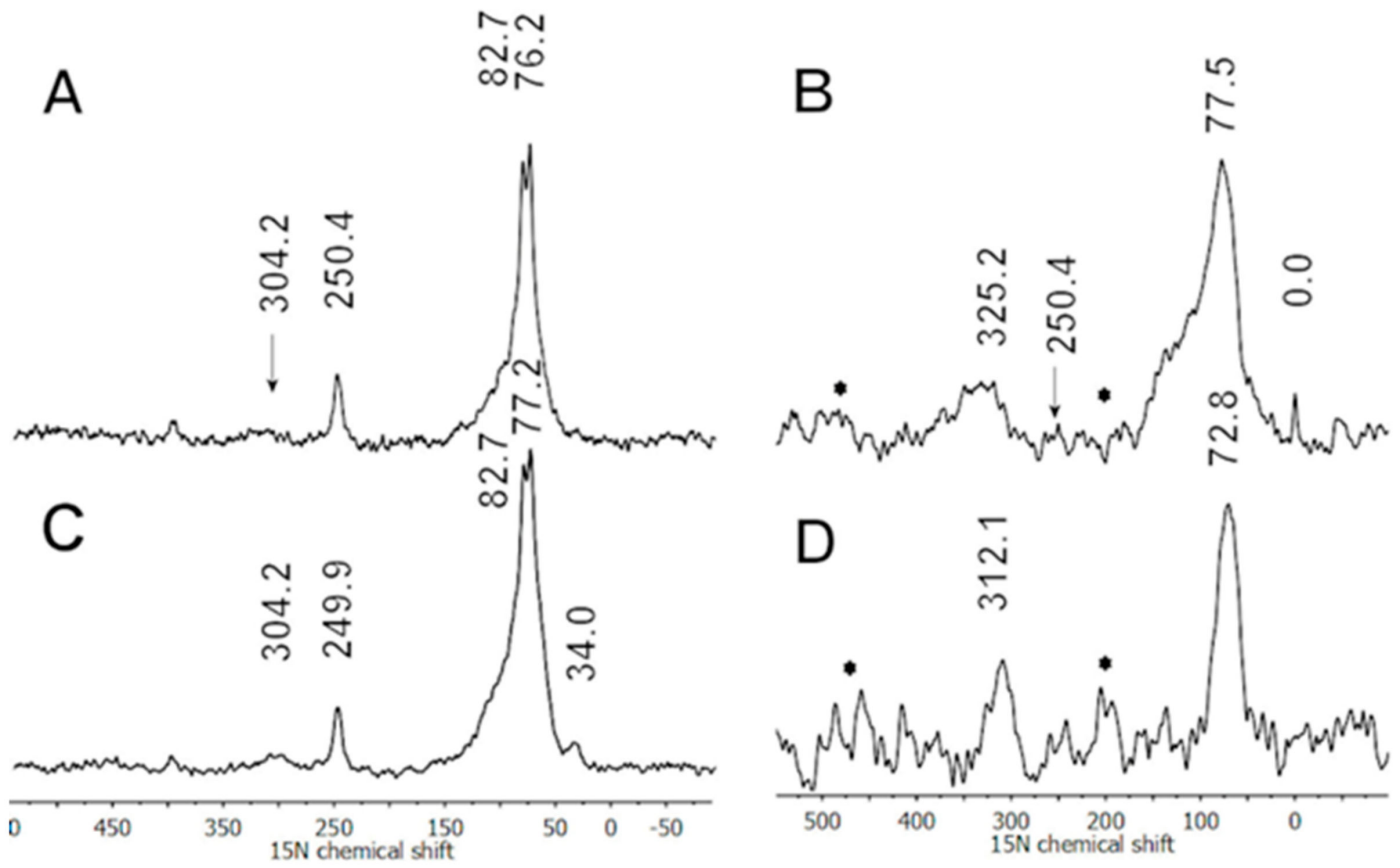
The solid-state ^15^N CP/MAS (**A**,**C**) and direct polarization (**B**,**D**) NMR spectra of the SD and DD sample, respectively, obtained after 24 h reaction time using ammonium persulfate without a dopant acid. Reprinted with permission from reference [17]. Copyright (2013) Elsevier.

It was observed that the ^15^N CP/MAS experiment did not match the results expected for PANI and its nanostructured products [16,19]. This was especially evident for the imine nitrogens which were often significantly underestimated in the ^15^N spectra [16,19]. It was thought that the nature of charge carriers and their locations in the nanostructured products could have significant roles in the detection of SSNMR signals. Therefore, Zujovic et al. explored the effects of polarons in the single (NH_4_OH, SD) and double (NH_4_OH + LiOH, DD) dedoped nanostructured products which were obtained in the oxidative polymerization of ^13^C/^15^N double-labeled PANI during the “pH falling” reaction [17]. ^15^N and ^13^C single pulse excitation (SPE) (direct polarization) and CP/MAS experiments were carried out. The effects of polarons were evidenced in the ^15^N CP/MAS spectra, (Figure 6A,C). The imine nitrogen was not detected at a significant level in both SD and DD CP/MAS spectra (Figure 6A,C) due to the presence of residual unpaired spins associated with the occurrence of positive charges (EPR data). At the same time the SPE spectra (Figure 5B,D) revealed both the imine and amine resonances. This implied that the CP/MAS method was more sensitive to the effects of positive charges. This finding was supported by ^13^C variable contact time experiments [17]. Following the second dedoping with LiOH positive charges were largely removed in the DD sample, which resulted in the decreased intensity of the shoulder at ~120 ppm (Figure 6B) and increased intensity of the imine resonance detected at ~312 ppm (Figure 6D) in the ^15^N SPE spectra. The EPR results showed more than an order of magnitude reduction in the number of unpaired spins after double dedoping. ^15^N SPE experiments confirmed that positive charges affect the quinoid part (imine) more than the benzenoid (amine) part [44]. The reason that nanostructured PANI was more difficult to dedope compared to its standard, less ordered counterpart was that the latter allowed a more random and effective interaction with a dedoping agent. 

Goddard et al. analyzed ^2^H (deuteron) MAS spectra of the conductive ring-deuterated PANI salts (ES/HCl). They were able to detect a Knight shift as a consequence of the presence of delocalized polarons by analyzing individual sidebands whose frequencies and widths are free from the quadrupole interaction in ^2^H MAS spectra [32]. The delocalized electron spins were located in both crystalline and amorphous parts, causing hyperfine coupling to deuterons. The sidebands in the ES spectrum consisted of two overlapping resonances, one at the same shift as the insulating EB form and the other was shifted by 5.8 ppm, compared to the position of the peak found in the spectrum of the EB form. The direction and the field and temperature independent magnitude of the shift confirmed the presence of the Knight shift. The authors also emphasized that in the highly conductive samples, the signal loss was caused by both dephasing (the proximity of unpaired, localized electrons) and high r.f. (radio-frequency) reflectance [32].

Giotto et al. used ^7^Li NMR spin-lattice relaxation measurements and linewidths analysis of the PANI films doped with LiClO_4_ at two different doping levels as a function of temperature. The temperature dependence of ^7^Li *T*_1_ relaxation was not in line with the BPP (Bloembergen-Purcell-Pound) model possibly due to the presence of polarons [71]. On the other hand, the higher doping promoted the faster ^7^Li *T*_1_ relaxation, again probably due to the higher concentration of polarons with unpaired electrons. At the same time, the glass transition temperatures and the ^7^Li *T*_1_ temperature dependence of the samples obtained at two different doping levels implied that the sub-chains were less mobile in the polymer doped at the higher concentration of LiClO_4_ [71]. This was based on the finding that the glass transition occurred at lower temperatures in the lower doped sample.

Kababya et al. used ^15^N, ^13^C CP/MAS and ^7^Li MAS experiments to characterize the PANI obtained in the presence of dodecylbenzene sulfonic acid (DBSA) [34]. They emphasized the necessity for using the LiOH for dedoping as the DBSA interacted strongly with the imine nitrogens. Dedoping of PANI-DBSA with NH_4_OH was incomplete due to the strong interaction of the imine groups and residual bipolaron states. The incomplete dedoping was detected in the ^15^N CP/MAS spectrum as a broad shoulder at 100–140 ppm. LiOH removed a significant part of the dopant acid, which was again confirmed in the ^15^N CP/MAS spectrum by the disappearance of the broad shoulder. To probe heterogeneity of the dedoped PANI-DBSA sample Kababya et al. carried out *T*_1ρ_^H^ measurements. The single exponential relaxation curve proved that DBSA was mixed with PANI at the molecular level. ^15^N{^7^Li} rotational-echo double resonance (REDOR) data of a LiOH dedoped sample suggested that there existed two imine-amine pairs A_1_-I_1_ and A_2_-I_2_ with the different binding strength to DBSA. The Li+ cations were near the A_2_-I_2_ pairs (a higher DBSA binding affinity), closer to the I_2_ than A_2_, i.e., next to the imine with the stronger DBSA binding, at a distance of ~4 Å.

Wehrle et al. used ^15^N CP/MAS and NQS experiments to study the structure and the role of charge carriers in electrochemically synthesized PANIs under various acidities [36,37]. They analyzed ^15^N PANI spectra and performed peak assignments using the model substances of aniline, N-phenylcyclohexanonimine and azophenine. In the spectrum of PANI, the authors assigned peaks at ~60 and ~320 ppm to amine and imine nitrogens, respectively. Wehrle et al. also analyzed the polymer obtained under highly acidic conditions. The ^15^N spectrum exhibited two resonances: a lower intensity one and at a lower field (~350 ppm) assigned to the imine nitrogen; the very broad one at a higher field (~100 ppm), attributed to the superposition of various structures in the presence of polarons (confirmed by EPR), bipolarons and also affected by spin dynamics as well as electron and nuclear motions. 

Sahoo et al. used ^15^N and ^13^C CP MAS to study PANI and PANI prepared by oxidative enzymatic polymerization with and without the presence of polyelectrolyte templates [72]. Their goal was to understand how enzymatic synthetic conditions using a natural or a synthetic biomimetic catalyst and the presence/absence of polyelectrolyte templates affected the charge distribution and structural heterogeneity of the final products. It was found that at the higher doping levels, after reaching the maximum, polaronic contribution started decreasing and the bipolarons became the main charge carriers. ^15^N CP MAS end-group studies revealed that it was possible to get information about the molecular weight of the enzymatically synthesized polymer. The *T*_1C_ relaxation measurements at different temperatures exhibited the linear dependence of 1/*T*_1C_ with temperature suggesting the Korringa-type relation for the *T*_1C_ relaxation mechanism usually seen in metals.

Syed et al. applied ^13^C CP/MAS experiments and analyzed the structure and doping process in PANIs obtained by a one-step redox polymerization of aniline with persulfate in varying molar ratios [73]. The authors compared the spectra of the doped ES and dedoped PANI samples. They made an important observation that, although the dedoping process involved the elimination of charges, the chemical structure remained the same [73]. 

Menardo et al. used ^13^C CP/MAS and NQS experiments to propose the coexistence of –NH^+^= units, obtained by protonation of imine nitrogens and –NH_2_^+^– units obtained by protonation of amine nitrogens at pH ≃ 0 in the doped form of PANI [74]. The authors confirmed that the reduced form of PANI (LEB) at pH ≃ 0 consisted of benzenoid rings and that about 25% of the amine nitrogens could be protonated to –NH_2_^+^. A proposal of the coexistence of the -NH+= and –NH_2_^+^– units had implications on the model of the conducting state in the doped PANI.

## 5. Conclusions

Solid-state NMR spectroscopy has been well established as a valuable and powerful analytical technique in polymer science. In this review, we have presented a summary of the standard and advanced multidimensional and multinuclear solid-state NMR (SSNMR) techniques that are used in characterizing structural and molecular dynamic features of the conducting polymer PANI and PANI based materials such as composites, complexes and copolymers. The single- and multidimensional ^13^C and ^15^N spectroscopies have been used for the analysis of the structural properties and the polymer backbone arrangements. It has been shown that ^2^H NMR can be used to analyze the mobility of the benzene rings, while ^13^C relaxation measurements revealed the molecular dynamics of the polymer backbones and side-chain structural units. The studies of the nature and distribution of charge carriers and their role in the conductivity of PANIs, mainly by ^15^N and ^2^H spectroscopies, have been reviewed. This paper has also presented various SSNMR studies on PANI related nanostructures and the role of various dopants on the structure and conductivity of PANI.
